# The C-Terminus of Perilipin 3 Shows Distinct Lipid Binding at Phospholipid-Oil-Aqueous Interfaces

**DOI:** 10.3390/membranes11040265

**Published:** 2021-04-06

**Authors:** Amber R. Titus, Ellyse N. Ridgway, Rebecca Douglas, Elena Sánchez Brenes, Elizabeth K. Mann, Edgar E. Kooijman

**Affiliations:** 1 Department of Biological Sciences, Kent State University, Kent, OH 44242, USA; eridgwa1@kent.edu (E.N.R.); rdougl11@kent.edu (R.D.); 2 Escuela de Física, Universidad de Costa Rica, San José 11501, Costa Rica; elena.sanchezbrenes@ucr.ac.cr; 3 Department of Physics, Kent State University, Kent, OH 44242, USA; emann@kent.edu

**Keywords:** lipid droplet, interfacial tension, amphipathic α-helix bundle, perilipins

## Abstract

Lipid droplets (LDs) are ubiquitously expressed organelles; the only intracellular organelles that contain a lipid monolayer rather than a bilayer. Proteins localize and bind to this monolayer as they do to intracellular lipid bilayers. The mechanism by which cytosolic LD binding proteins recognize, and bind, to this lipid interface remains poorly understood. Amphipathic α-helix bundles form a common motif that is shared between cytosolic LD binding proteins (e.g., perilipins 2, 3, and 5) and apolipoproteins, such as apoE and apoLp-III, found on lipoprotein particles. Here, we use pendant drop tensiometry to expand our previous work on the C-terminal α-helix bundle of perilipin 3 and the full-length protein. We measure the recruitment and insertion of perilipin 3 at mixed lipid monolayers at an aqueous-phospholipid-oil interface. We find that, compared to its C-terminus alone, the full-length perilipin 3 has a higher affinity for both a neat oil/aqueous interface and a phosphatidylcholine (PC) coated oil/aqueous interface. Both the full-length protein and the C-terminus show significantly more insertion into a fully unsaturated PC monolayer, contrary to our previous results at the air-aqueous interface. Additionally, the C-terminus shows a preference for lipid monolayers containing phosphatidylethanolamine (PE), whereas the full-length protein does not. These results strongly support a model whereby both the N-terminal 11-mer repeat region and C-terminal amphipathic α-helix bundle domains of perilipin 3 have distinct lipid binding, and potentially biological roles.

## 1. Introduction

Lipid droplets (LDs) are highly complex, dynamic organelles that are critical for cellular energy regulation. These organelles are found in almost all cell types but are most prominent in adipocytes. Size, distribution, along with lipid and protein composition of each LD differs by cell type. LDs are similar in structure to extracellular lipoprotein particles, with a neutral lipid core (tri-, di-, monoacylglycerols and sterol esters) surrounded by a phospholipid monolayer [1,2]. The specific composition of this hydrophobic core depends on the specialized function of the cell type, e.g., stellate cells contain LDs filled with retinyl esters [3,4]. The LD core and monolayer composition are complex and not well established due to the difficulty in isolating individual LDs [2,5,6,7,8,9], and the observation that there are distinct LD populations in a single cell [10,11]. The consensus appears to be that the LD monolayer contains predominantly phosphatidylcholine (PC), but significant amounts of phosphatidylethanolamine (PE) and minor populations of other lipids are observed as well [2,5,6,7,8,12,13,14].

LDs form from the endoplasmic reticulum (ER) where neutral lipids are synthesized; lens-like structures (~40–60 nm) begin budding from the ER outer leaflet [15]. This lens formation is highly sensitive to the ER membrane composition and associated proteins, specifically seipin and promethin [16,17,18]. A new and compelling LD biogenesis model suggests that LDs form via liquid-liquid phase separation (LLPS), with the nucleated fat lens viewed as the condensed phase, the free neutral lipids within the ER as the diluted phase, and the ER membrane as the solvent [19,20]. This framework allows the budding of LDs to be driven by equilibrium concentration of triglycerides because the ER membrane environment and surrounding proteins alone cannot fully explain the energetics of triglyceride accumulation. Triacylglycerol and sterol esters are formed via acylation and disperse between ER leaflets until they reach a critical concentration and begin to bud from the ER outer leaflet [15,17,21,22]. After biogenesis, some LDs stay close to the ER and “dock” onto the ER membrane through an unknown mechanism, but most disperse throughout the cell via a non-random process [17,23]. Due to LD structural complexity and heterogeneity within a cell, it is critical to gain an understanding of how individual lipid components, such as lipid monolayer composition and physicochemical properties, affects LD function and its ability to recruit binding proteins.

LD binding proteins can be divided into two general classes: Class I and Class II. Class I LD binding proteins originate from the ER and contain a hairpin structure plus a positively charged domain [24]. Class II LD binding proteins typically contain more complex physical structures (e.g., amphipathic α helices) and are recruited from the cytosol to the LD surface. Some of these Class II proteins stay closely associated to LDs while others exchange between LDs and other organelles or cytosol throughout the cell. The Class II LD binding proteins have many similarities to apolipoproteins [24,25,26,27,28,29]; for example, both contain long amphipathic α-helices, some of which form helix bundles that are soluble in solution as found in perilipin 3 and apoE [30]. Apolipoproteins are generally divided into two groups: non-exchangeable and exchangeable. Non-exchangeable, or lipid-bound apolipoproteins, are most similar to the Class II LD binding proteins that stay bound to the lipoprotein particle. Exchangeable apolipoproteins like apoE exchange between the blood and the lipoprotein particle and are similar to exchangeable Class II LD binding proteins that exchange between the cellular cytosol and LDs [31].

The perilipins are a family of LD binding proteins that are increasingly scrutinized due to a possible connection between perilipin-mediated lipid metabolism and whole-body metabolism [32]. This family contains five mammalian members (perilipin 1–5) and is the most abundant LD-associated protein family expressed in humans [33,34]. This family of proteins is formed on free ribosomes in the cellular cytosol, and some members are known to be stable in aqueous environments [25,34,35]. An amphipathic α-helix bundle is found at the C-terminus of perilipins 2, 3, and 5. Amphipathic α-helix bundles function by concealing hydrophobic protein residues from the aqueous cell environment. This α-helix bundle has a resemblance to the lipid binding domains of the well-characterized apolipoproteins, apoE and apoLp-III [36,37,38,39]. Unlike for apoLp-III, this C-terminal amphipathic α-helix bundle does not appear to be required for LD targeting. In addition, also present in these proteins is an 11-mer repeat region in the N-terminus, which may also form amphipathic α-helices. It has been well established that this region alone is sufficient for LD targeting and binding [23,25,29,34,40,41,42,43]. Interestingly, both the C-terminus of perilipin 3 and full-length protein behave like apoE in DISC assays, leading to the hypothesis that perilipin 3 may have apolipoprotein-like properties in vitro and in vivo [42]. This behavior suggests that this amphipathic α-helix bundle may also be involved in lipid binding but its exact function in cells has not been elucidated to date.

We reported in Mirheydari and Rathnayake et al. 2016 that perilipin 3’s truncated C-terminal α-helix bundle shows greater insertion into lipid monolayers at the air-aqueous interface compared to the full-length protein [44]. We also found that the C-terminus of perilipin 3 showed preferable insertion to 1-palmitoyl-2-oleoyl-sn-glycero-3-phosphocholine (POPC) compared to 1,2-dioleoyl-sn-glycero-3-phosphocholine (DOPC). In this study, we expand our previous work on perilipin 3 with a more physiologically relevant model system. Pendant drop tensiometry has been used to characterize multiple apolipoproteins and LD binding proteins [11,23,44,45,46,47], but overall is an under-utilized technique in the field of protein-lipid interactions. Here, we use pendant drop tensiometry to characterize full-length perilipin 3 and a C-terminal truncation at oil-phospholipid-aqueous interfaces. We find that the C-terminus of perilipin 3 is highly surface active, with a preference for unsaturated lipids at the oil-aqueous interface. We find that adding PE increases the affinity of the C-terminus of perilipin 3 to the phospholipid-oil interface. The full-length protein does not show this PE dependence. We also show here that the C-terminus of perilipin 3 has distinct lipid binding compared to the 11-mer repeat region. We propose that the C-terminal amphipathic α-helix bundle of perilipin 3 may help to “anchor” the protein to LDs after initial localization from the N-terminal 11-mer repeat region.

## 2. Materials and Methods

### 2.1. Protein Purification

Full-length (amino acids 1–434, PLN3A) and truncated (amino acids 187–434, PLN3D) perilipin 3 constructs were prepared and stored as described in Mirheydari and Rathnayake et al. 2016 [44]. SDS-PAGE gels were used to check protein expression at each chromatography step. Protein concentration was checked with Nanodrop 1-position spectrophotometer (ND-2000) and constructs were sent to the Learner Research Institute Proteomics Laboratory (Cleveland Clinic Foundation) for sequencing via LC-MS/MS. All constructs were found to be suitably pure (>85%) for biophyisical characterization.

### 2.2. Buffer Preparation

The buffer used for all experiments was prepared with 150 mM KCl, 10 mM Tris, 0.25 mM EDTA, 1 mM KOH (all >99% purity, Sigma Aldrich) in HPLC-grade water, pH adjusted to 7.20 ± 0.05. Salts were treated via heating at 100 °C under vacuum for at least 24 hrs before use [45]. The buffer is kept in the experimental room at 21 ± 0.1 °C to minimize density variations. The density of three batches of buffer made on different days were measured using a DE45 Delta Range Density Meter (Mettler Toledo) and were found to be within 0.001 g/cm^3^. To ensure minimal contamination of surface active components, fresh buffer was made at least once a week.

### 2.3. Vesicle Formation

All phospholipids were purchased from Avanti Polar Lipids (Alabaster, AL, USA). Triolein was purchased from Nu-Chek-Prep (Elysian, MN, USA). Pure lipid was dissolved in 2:1 chloroform:methanol (>99% purity, Thermo Fisher Scientific, Waltham, MA) at a concentration of ~0.1 mM to prepare lipid stocks. Lipid films were made in a borosilicate glass tube by drying a specific volume of lipid stock solution(s) under a stream of nitrogen. The films were kept under vacuum overnight to remove residual traces of organic solvent and stored at −20 °C. Lipid films were rehydrated with 4 mL HPLC-grade water and vortexed for ~30 s. After vortexing, the mixture was put through five rounds of rapid freeze-thaws. This mixture was then extruded through a 200 nm and 100 nm filter following standard procedure (T&T Scientific, Knoxville, TN, USA). The size of the resulting vesicles was measured using DLS (differential scanning calorimetry, Horiba DLS 7100, SZ-100 series) to be between 50–250 nm. We found no significant difference between lipid adsorption to the oil surface with these size differences.

### 2.4. Pendant Drop Tensiometer Setup

The pendant drop tensiometer setup consists of borosilicate glass cuvette, a 100 μL Hamilton syringe held vertically by a Legato 130 programmable syringe pump from KD Scientific fixed to a stainless-steel stand, a Pixelink PL-B776F CCD camera, a Thorlabs, OSL1 High Intensity Fiber Illuminator light source and a glass diffuser. All units are mounted on a linear rail on a Kinetic Systems, Vibraplane 5720E-3036-21 vibration free table. This system is stored in a temperature-controlled room and each experiment was conducted at 21 ± 0.1 °C. Before each experiment, the enclosure was wiped with methanol to remove any dust. The syringe and cuvette were each cleaned with KOH solution consisting of 24 g of pure water, 24 g of KOH, and 164 g of ethanol, followed by at least three rinses with deionized water and finally with three rinses of HPLC-grade water, and then left to dry completely in a clean environment at 21 °C.

### 2.5. Lipid Adsorption Protocol

**Figure 1:**
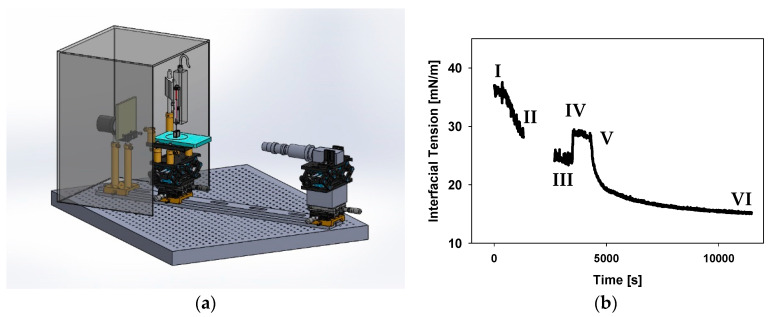
(**a**) Model of the pendant drop tensiometer setup used for all data collection. (**b**) Typical data for a full lipid adsorption, expansion of lipid monolayer, and interaction with protein experiment. In I, a 5 μL triolein drop in buffer is formed and left to equilibrate. During time II, a 1-palmitoyl-2-oleoyl-sn-glycero-3-phosphocholine (POPC) monolayer is formed. The gap in data between II and III indicates the time where residual vesicles are removed. At III, the lipid monolayer is expanded by increasing the oil drop volume. In IV, the expanded drop monolayer was left to equilibrate and then protein (either full-length or C-terminal construct) was added and allowed to insert V-VI.

An example of one full experiment is shown in Figure 1 with numbered steps. Corresponding numbers are listed in the following description. A clean Hamilton syringe is filled with fresh triolein at 21 °C, after which the straight needle is replaced with a custom-made J-shaped needle. The syringe is placed into the syringe pump holder and wiped down with methanol. The syringe is lowered into a freshly-filled cuvette containing 10 mL of buffer. A drop of either 5, 10, or 15 μL is formed at a rate of 1 μL/s. After 5–10 min of droplet equilibration I, 4 mL of buffer is carefully removed from the cuvette and replaced with the lipid vesicle suspension to a final concentration of 0.115 mM. After ~30 min of mixing (allowing the lipids to fully adsorb to the triolein interface), II, the buffer in the cuvette is serially diluted with at least 40 mL of fresh buffer to remove unbound lipid. Note, because the influx/efflux of buffer causes droplet movement, images are recorded but not analyzed during this buffer flush. After the buffer flush, the drop has another 5–10 min equilibration period with its newly formed lipid monolayer, III. The size of the drop is either increased or decreased to alter lipid packing at a rate of 0.1 μL/s, IV. Following the expansion/contraction of the drop and another 5–10 min equilibration period, the protein of choice (either the C-terminus of perilipin 3 or full-length perilipin 3) is added to the cuvette, V, to a final concentration of 0.15 μM for 2 h, VI.

### 2.6. Axisymmetric Drop Shape Analysis (ADSA)

The shape of the pendant drop is reliant on the balance between gravity and surface or interfacial tension. The interfacial tension makes the drop more spherical while gravity elongates the drop. By analyzing the silhouette of the drop through time, we gain accurate measurements of the interfacial tension of a given system. We take images of the triolein drop in buffer every 5 s through the entirety of an experiment. Each experiment produces approximately 2000 images; we use axisymmetric drop shape analysis (ADSA) software developed by the Neumann Lab in Toronto to run the interfacial tension calculations needed [48]. ADSA provides estimates of interfacial tension based on an optimized fit of the silhouette of a fluid droplet, determined using the CANNY algorithm [49], to the Young–Laplace equation of capillarity [50,51]:

$$\Delta P=\gamma \left(\frac{1}{R_{1}}+\frac{1}{R_{2}}\right)=\Delta P_{0}+\left(\Delta \rho \right)gz$$

where, ΔP refers to the Laplace, or capillary, pressure across the surface of the drop at any point; γ represents the droplet interfacial tension; Δρ is the density difference between the triolein and buffer; $\frac{1}{R_{1}}$ and $\frac{1}{R_{2}}\;$are the principal radii of curvature at the point; g is the gravitational acceleration; z is the distance along the axis of symmetry between the point and a reference point where the pressure difference is $\Delta P_{0}$.

The reproducibility of the interfacial tension values for each droplet, which depends on both the experimental set-up and the physical chemistry of all components, was found to be ≤ 3.3 mN/m. This pendant drop tensiometer does not measure the interfacial tension directly, but rather the capillary length,$\;\lambda_{c}$, which is defined by:

$$\lambda_{c}={\left(\frac{\gamma }{g\Delta \rho }\right)}^{1/2}$$

The uncertainty in the interfacial tension is thus given by:

$${\left(\frac{\delta \gamma }{\gamma }\right)}^{2}={\left(\frac{\delta \lambda_{c}{}^{2}}{\lambda_{c}{}^{2}}\right)}^{2}+{\left(\frac{\delta \left(\Delta \rho \right)}{\Delta \rho }\right)}^{2}$$

ADSA can be applied only to well-deformed droplets, which is quantified by calculating dimensionless Neumann numbers (Ne) [52]:

$$Ne=\;\frac{\Delta \rho gR_{0}H}{\gamma }$$

**Figure 2:**
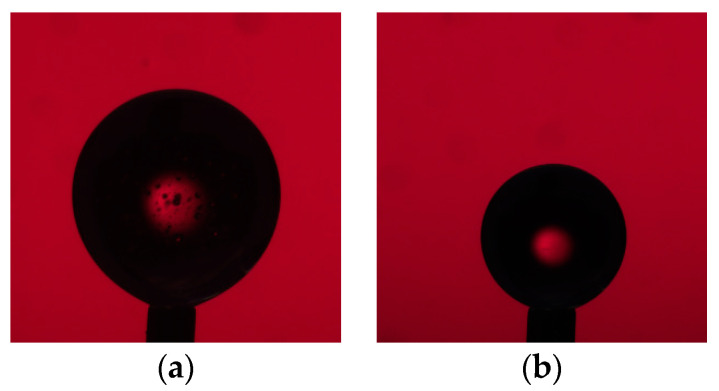

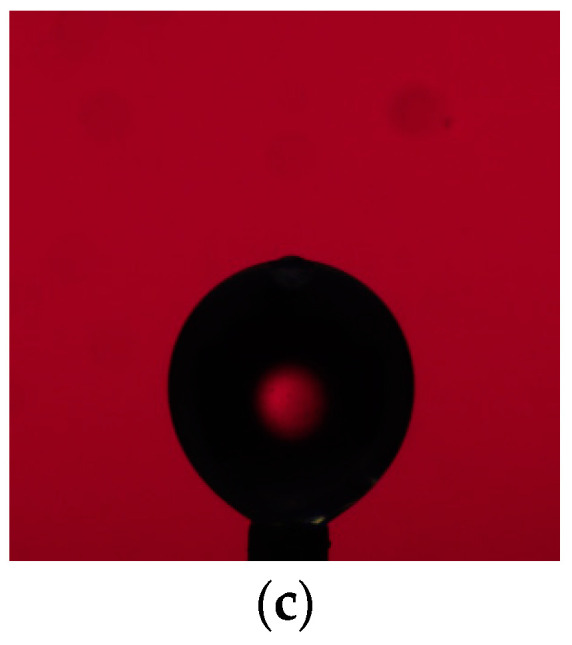
Examples of typical droplet images taken for interfacial calculation by axisymmetric drop shape analysis (ADSA): (**a**) 15 μL neat triolein, Ne=0.8; (b) 5 μL neat triolein, Ne=0.5; (c) 5 μL triolein coated in POPC and full-length perilipin 3, Ne=0.7.

In this equation, $R_{0}$ is the radius of curvature at the drop apex and H is the drop height. Generally, larger drops will have more deformed (elongated) shape, while smaller drops tend to be more spherical. For stationary, uncoated droplets of triolein with relatively large interfacial tension, approximately 20 μL is ideal. The addition of surface-active components (e.g., lipids and proteins) increases the likelihood of droplet break-off so that the maximum droplet size is 15 μL. We find that drops of triolein in the size range 10–15 μL yield sufficiently deformed drops (Ne≥0.6) and thus accurate ADSA results (Figure 2a). For experiments where we alter π_Lipid_, we need to start with smaller droplets (approximately 5 μL) in order to ensure the droplet stability during and after expansion. When a lipid monolayer is added to a large drop and that drop is compressed, we find the drop to be sufficiently deformed. We also find sufficient deformation for small drops after the addition of a lipid monolayer compared to neat oil drops of the same size (Table 1). We find that uncoated, 5 μL triolein droplets are not elongated enough to provide accurate ADSA results compared to drops of the same size after the addition of lipid/protein (Figure 2b,c). Because of this, we measured the interfacial tension of pure triolein in 150 mM KCl buffer using three separate 15 μL drops, 38.3 ± 2.4 mN/m, and used this value as the initial interfacial tension for every experiment involving drop expansion or contraction.

## 3. Results

### 3.1. The C-Terminus of Perilipin 3 Is Surface Active at the Oil-Aqueous Interface

**Figure 3:**
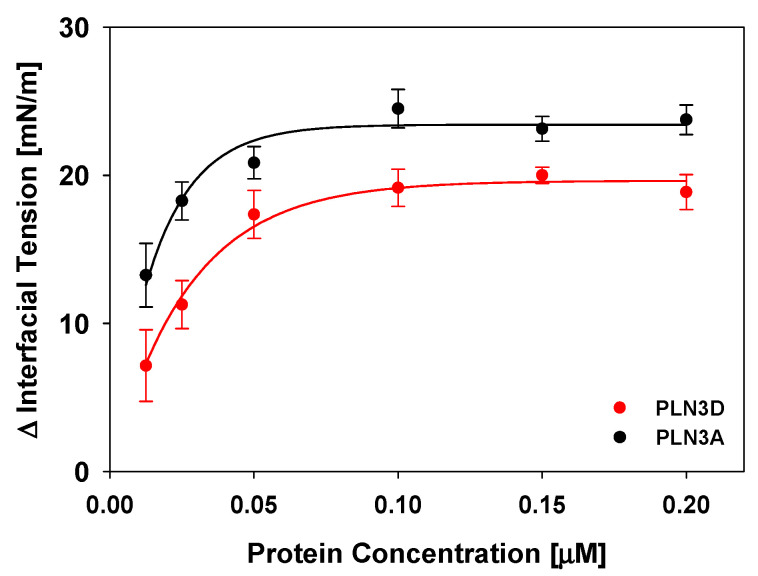
Surface pressure of the C-terminus of perilipin 3 (PLN3D, red) and full-length perilipin 3 (PLN3A, black) at the oil-aqueous interface. Each point is the average of three independent experiments in which a triolein drop is formed in buffer and allowed to equilibrate before either the full-length or truncated perilipin 3 is added at a set concentration and allowed to insert. Values reported here are the change in interfacial tension between the initial triolein in buffer value and after protein is added. Error bars are the standard deviations calculated between the three independent drops.

The surface activity of the C-terminal amphipathic α-helix bundle of perilipin 3 (aa 187–434) and full-length perilipin 3 (aa 1–434) was determined at the oil-aqueous interface (Figure 3). We find that both constructs are surface active at the oil-aqueous interface. The reduction in interfacial tension (γ) for both the full-length and truncated perilipin 3 constructs are higher at the oil-aqueous interface than what we reported at the air-aqueous interface (a reduction in γ of 53–69% compared to 15–26% [44]). The data in Figure 3 show that a concentration of 0.01 μM for the full-length protein is sufficient to fully maximize surface pressure (π = 23.1 ± 0.8 mN/m) at this interface, whereas a higher concentration, ~0.15 μM, is required for the C-terminus (π = 20.1 ± 0.5 mN/m). We used a protein concentration of 0.15 μM for all experiments to maximize interaction with the lipid monolayers investigated for both constructs.

### 3.2. A Fully Unsaturated PC Monolayer Allows for Greater Protein Insertion for Both Full-Length Perilipin 3 and Its C-Terminus at the Oil-Lipid-Aqueous Interface

Next, we tested the insertion of the C-terminal amphipathic α-helix bundle of perilipin 3 and the full-length protein with model lipid monolayers at the oil-lipid-aqueous interface. Unlike the well-studied cellular bilayer, the composition of the lipid monolayer covering LDs is currently not well understood. To model LD systems, it is thus crucial to vary lipid head group and acyl chain composition to systematically alter the physicochemical properties of the phospholipid monolayer. Previously, at the air-aqueous interface, we showed that perilipin 3 preferred lipids with more ordered acyl chains. Hence, we chose 1,2-dioleoyl-sn-glycero-3-phosphocholine (DOPC) and 1-palmitoyl-2-oleoyl-sn-glycero-3-phosphocholine (POPC) for our initial investigation. DOPC has two unsaturated (18:1 Δ9) acyl chains, whereas POPC has both a saturated (16:0), and an unsaturated (18:1 delta 9) fatty acid.

**Figure 4:**
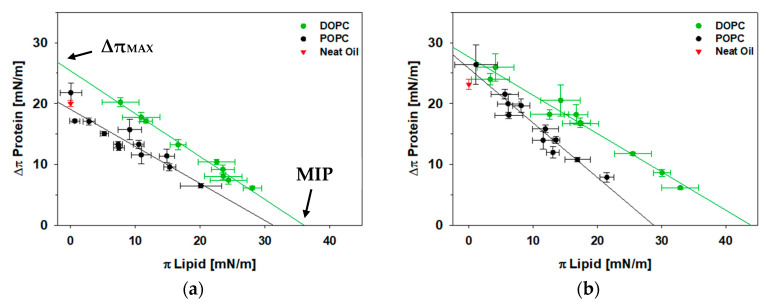
(**a**) Insertion isotherms of the C-terminus of perilipin 3 (PLN3D) as a function of lipid packing for 1,2-dioleoyl-sn-glycero-3-phosphocholine (DOPC, green) and POPC (black) monolayers; (**b**) Insertion pressure of full-length perilipin 3 (PLN3A) as a function of lipid packing for DOPC (green) and POPC (black) monolayers. Red triangles correspond to the protein surface pressure without lipid monolayer, 20.1 ± 0.5 mN/m and 23.1 ± 0.8 mN/m, for the C-terminus of perilipin 3 and the full-length protein respectively. Error bars are the standard deviation of surface pressure values after drop expansion/contraction (π_Lipid_) and after addition of protein (Δπ_Protein_).

Figure 4a,b shows the insertion data for the C-terminus of perilipin 3 and the full-length protein with monolayers of DOPC and POPC. Each point on the graph is one pendant drop experiment as described in the Methods section. The change in surface pressure of the lipid monolayer after expanding or compressing the drop size is plotted on the *x*-axis as π_Lipid_. The change in surface pressure of the lipid monolayer after the addition of either protein construct is plotted on the *y*-axis as Δπ_Protein_. Two key quantities from these insertion isotherms to take note of are the estimated maximum insertion pressure (MIP) and the maximum change in monolayer pressure (Δπ_MAX_). MIP (the x-intercept), or exclusion pressure, is the surface pressure above which the protein is no longer able to insert into the monolayer [53]. Unlike at the air-aqueous interface, we observe here that at the oil-lipid-aqueous interface a fully unsaturated monolayer allows for a significantly greater protein insertion, corresponding to a MIP increase of ~18% and ~30%, for the C-terminus of perilipin 3 and the full-length construct respectively (Figure 4).

### 3.3. At the Oil-Aqueous Interface, Addition of POPE Increases Insertion of the C-Terminus of Perilipin 3, But Not for the Full-Length Protein

**Figure 5:**
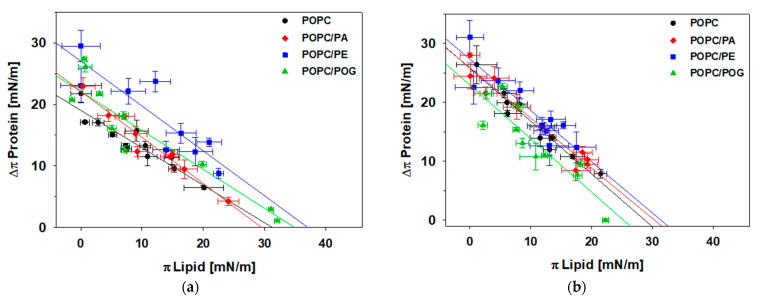
Insertion isotherms for: (**a**) The C-terminus of perilipin 3 (PLN3D) as a function of lipid packing for POPC (black circle), 7:3 POPC/POPA (red diamond), 7:3 POPC/POPE (blue square), and 7:3 POPC/POG monolayers (green triangle); (**b**) full-length perilipin 3 (PLN3A) as a function of lipid packing for POPC (black circle), 7:3 POPC/POPA (red diamond), 7:3 POPC/POPE (blue square), and 7:3 POPC/POG monolayers (green triangle). Error bars are the standard deviation of surface pressure values after drop expansion/contraction (π_Lipid_) and after addition of protein (Δπ_Protein_).

Next, we investigated the insertion of the C-terminus of perilipin 3 and the full-length protein in mixed lipid monolayers. It is well documented that lipids with negative spontaneous curvature in cell/organelle membranes help to facilitate binding of peripheral proteins due to increased access of hydrophobic protein domains to the hydrophobic acyl chains of the lipids [54,55]. Previous data from our lab showed that this may occur at the LD monolayer as well, with lipids of negative curvature stress (1-palmitoyl-2-oleoyl-sn-glycero-3-phosphoethanolamine (POPE), 1-palmitoyl-2-oleoyl-sn-glycero-3-phosphate (POPA), 1-palmitoyl-2-oleoyl-sn-glycerol (POG)) allowing for increased perilipin 3 insertion [44]. Figure 5a,b, along with Table 2 and Table 3, show the insertion of the C-terminus and full-length protein in POPC monolayers containing 30 mol% of POPE, POPA, or POG. The full-length protein shows no significant difference in its insertion behavior with the addition of other lipid species. However, the C-terminal domain of perilipin 3 has a higher Δπ_MAX_ and MIP for the POPE-containing lipid monolayer, suggesting that lipids with negative spontaneous curvature influence its monolayer insertion. The difference between Δπ_MAX_ values for the C-terminal domain with pure POPC and with POPE-containing monolayers are statistically significant, with the difference in MIP values being less obvious (Table 2). This insertion is also higher than that observed for the C-terminus alone at a neat triolein surface (20.1 ± 0.5 mN/m).

In previous work, we showed that the negative charge of PA increased the affinity of apolipoproteins to the oil-lipid-aqueous interface [44,46]. Here we show that perilipin 3 recruitment is largely unaffected by 30 mol% POPA. We observe no significant difference in Δπ_MAX_ and MIP for the C-terminal domain and full-length perilipin 3 compared to just a POPC monolayer. This data suggests that negative charge negates the effect of negative spontaneous curvature in the process of recruitment and insertion of perilipin 3. We also investigated the effect of the diacylglycerol POG on perilipin 3 recruitment and insertion. Interestingly, we observe no difference in Δπ_MAX_ and MIP (see Figure 4, green data points).

## 4. Discussion

The recruitment and insertion of LD binding proteins is critical to the biogenesis and function of LDs, but this process is not fully understood. To date, there are very few publications detailing the in vitro interaction of LD binding proteins with relevant LD mimicking model systems [11,23,45,46,47,56]. Previous work conducted by multiple groups has concluded that for perilipin proteins, the N-terminal 11-mer repeat region is the LD targeting and binding domain [23,41,42,43]. We previously showed, at the air–water interface, that in the context of the full-length protein, the C-terminus of perilipin 3 appears to not interact with the lipid monolayer. However the C-terminus alone showed strong insertion into phospholipid monolayers at the air-water interface [44]. How, and if, this C-terminal amphipathic α-helix bundle, present in perilipins 2, 3, and 5, assists in LD localization and binding has been ambiguous. The effect of LD monolayer lipid composition on the binding of specific domains of perilipin 3 was unknown. Here, we use perilipin 3 to shed new light on the interaction of this C-terminal domain with physiologically relevant model LD systems.

### 4.1. Lipid Acyl Chain Unsaturation Assists in Perilipin 3 Binding and Monolayer Insertion at the LD Interface

Recent molecular dynamics simulation data suggests that there may be “gaps” present in LD monolayers, which exposes the internal hydrophobic core of LDs to LD-binding proteins [57,58]. Interdigitation of neutral oil with the phospholipid monolayer of LDs may assist in the binding of specific protein domains, or amino acid residues. An increase in phospholipid unsaturation may allow for more fluidity in the LD monolayer and thus more triolein interdigitation due to matching of the oleic acid acyl-chains in the PC monolayer and triacylglycerol interior of the LD.

At the air-water interface, both the full-length protein and the C-terminal domain of perilipin 3 showed greater insertion into more saturated POPC monolayers [44], opposite of what we find here. Missing in the Langmuir monolayer system at the air-water interface is the oil (triolein). Our observations here thus suggest that perilipin 3 interacts significantly with the triolein, possibly through triolein interdigitation in a more fluid monolayer. The importance of the triolein core further supports pendant drop tensiometry as a more relevant model system to study LD-protein interactions.

We also show that Δπ_MAX_, which is a measure of protein monolayer affinity, for the DOPC monolayer, is significantly higher than Δπ for the protein on the oil interface alone (~6 mN/m for C-terminus and ~7 mN/m for full-length perilipin 3, indicated by the red data points in Figure 4). This means that perilipin 3 binding, for both the C-terminus and the full-length protein, is cooperative [45] for the DOPC monolayer. However, the C-terminus does not appear to show cooperative insertion for the POPC monolayer. The lipid monolayer with DOPC but not with POPC facilitates protein binding and insertion at the oil-phospholipid interface. This contrasts with our previous work on the α-helix bundle domains of the apolipoproteins apoE 3, and apoLp-III which do not show significant cooperativity [46] with either partially or fully unsaturated lipids. From the data presented here, we cannot exclude the possibility that acyl chain length also plays a role in perilipin-lipid interaction. No such specificity has been observed for perilipin 3 or other perilipins, but future studies may be warranted.

### 4.2. PE Facilitates Recruitment of the C-Terminal α-Helix Bundle of Perilipin 3 to LDs, But Not the Full-Length Protein

Addition of 30 mol% POPE to a POPC monolayer increases Δπ_MAX_ (~10 mN/m), and MIP (~8 mN/m) for the amphipathic α-helix bundle domain. In contrast, full-length perilipin 3 does not discriminate between phospholipids with the same acyl-chain composition but varying lipid head group. This observation suggests that, in the context of the full-length protein, the C-terminal domain of perilipin 3 may not interact directly with the phospholipid monolayer at the lipid-oil interface. This would follow our previous results in Langmuir monolayers at the air-water interface where we observed that the C-terminus had a significantly higher MIP than the full-length protein, clearly suggesting that the C-terminus was not involved in lipid monolayer binding and insertion in that specific case [44]. However, since the full-length protein has a higher Δπ_MAX_ and MIP at the lipid-oil interface, just no PE dependence, it is possible that the C-terminus is still involved in LD recognition and initial binding. Nevertheless, in the context of the full-length protein no final effect of lipid head group is observed.

PE is a lipid with negative spontaneous curvature, and membrane binding proteins are well known to bind better to the lipid bilayer as a function of increasing negative (spontaneous) curvature [54,55,59,60]. Indeed, we also observed higher MIP values for lipids with negative spontaneous curvature at the air-water interface for the C-terminus of perilipin 3 [44]. In the case of the full-length protein, this effect was significantly reduced [44]. Negative curvature increases the accessibility of hydrophobic protein domains or amino acid residues to the hydrophobic interior of a membrane. At the oil-lipid interface, it is likely that a similar scenario unfolds. At the oil-lipid interface, PE may also facilitate triolein interdigitation into the lipid monolayer, something that should be explored further with MD simulations [57,58]. PE allows the C-terminal domain to more easily reach the oil. It is unclear why we do not observe the same effect for the full-length protein, but it may be related to the distribution of large hydrophobic amino acids and the amphipathicity of the interacting amphipathic α-helixes between both domains [56]. What is clear is that the C-terminus of perilipin 3 shows significantly more cooperative binding and insertion then the full-length protein. Hickenbottom et al. showed that in the crystal structure of the C-terminal domain of perilipin 3 a hydrophobic cleft is present between the helix bundle, and the so called α/β domain N-terminal to the helix bundle [30]. This hydrophobic cleft is lined by several large hydrophobic residues (W and F) that may drive PE sensitivity of this domain.

Recently, PE was suggested to facilitate lipid droplet binding of perilipin 2 [61]. However, this is the first observation of PE mediated lipid binding for perilipin 3. Our results suggest that the C-terminus of perilipin 3 is selectively recruited to LD monolayers containing PE. Whether the C-terminal amphipathic α-helix bundle present in perilipin 5 shows similar affinity for lipid monolayers containing PE is still unknown. Future work on exchangeable perilipins should explore lipid monolayer insertion specificity in vitro using the pendant drop model system to gain further insight into how LD binding proteins target, and bind, LDs in vivo.

It should be noted that while we did not observe a significant effect of the diacylglycerol POG (a lipid with strong negative spontaneous curvature [62,63]) on binding and insertion of perilipin 3 this may have been caused by an experimental artifact. Previously, we observed that liposomes containing significant amounts (> 15 mol%) of diacylglycerol do not form normal single bilayer structures [59]. Instead, these liposomal dispersions contain at least 25% of liposomes with massive amounts of internal membranes. It is thus possible that the concentration of POG on the model LD interface was significantly lower than the 30 mol% of PE and PA. Future experiments utilizing less diacylglycerol will clarify this issue.

Perilipin 3 recruitment to LDs is not driven by negative charge as we observe no effect of the addition of 30 mol% POPA to our POPC monolayers. This is in contrast to our results with the apolipoproteins, apoE 3 and apoLp-III, which showed a significantly higher Δπ_MAX_ for PA containing monolayers. The C-terminal α-helix bundle domain of perilipin 3, like that of apoE 3, has positive charge, but we do not observe any effect on Δπ_MAX_. However, our C-terminal construct also contains the α/β domain as found in the crystal structure [30] which contains 9 anionic, and only 3 potential cationic residues. Hence the C-terminal domain contains significant negative charge unlike the apolipoproteins that we studied previously. Additionally, we found that apoE and apoLp-III do not show cooperative binding to a PC monolayer. In fact, PC significantly impedes LD monolayer binding for both amphipathic helix bundles of apoE and apoLp-III. The C-terminus of perilipin 3, in contrast, shows cooperative binding.

### 4.3. Proposed Model of Perilipin 3 Recruitment to Nascent LDs

Our data suggests that full-length perilipin 3 overall has higher levels of association with oil-phospholipid monolayers, but under specific conditions, the C-terminus of perilipin 3 shows distinct binding and insertion behavior. This work thus underscores the importance of investigating the different domains of perilipins, and hints at a unique biological function for the α-helix bundle domain. Perilipin 2 and 3 are well documented as binding to nascent LDs [17,40,42]. In this process, both ER phospholipid unsaturation and PE accumulation, have been shown to facilitate the nucleation of triglycerides within the ER bilayer [64]. Recently, a model of “hierarchical” binding was proposed for perilipins 1–3, with perilipins 2 and 3 being displaced by perilipin 1 as LDs mature [41]. Such a model should consider the physicochemical differences in lipid content on LD monolayers and the effect this would have on protein recruitment and insertion. Perilipin 2 and 3-containing LDs have phospholipid monolayers with higher levels of unsaturation compared to perilipin 1-containing LDs [8], consistent with our results for perilipin 3.

We propose that ER phospholipid unsaturation and PE accumulation may help recruit perilipin 3 to budding LDs during their formation via the C-terminal helix bundle domain. This contrasts with the proposal that the N-terminus of perilipin 3 is the region that localizes and initially binds to LDs in vivo [23,41]. This is difficult to verify using in vitro techniques because, while the C-terminal domain can be readily expressed and purified, the N-terminal 11-mer repeat region cannot [30,42]. However, the hydrophobicity of the 11-mer repeat region compared with that of the C-terminal domain (see supplementary information for the determination of amphipathic α-helices in the N-terminus using PSI-blast based secondary structure PREDiction (PSIPRED), and their calculated hydrophobicity compared to those for the helices in the helix bundle domain) do not show striking differences that would support the in vivo data. One possibility is that the in vivo results on perilipin 3 recruitment and binding to LDs is skewed by experimental conditions. Targeting of LDs is tracked via green fluorescent protein (GFP) fusion proteins which may lead to LD binding artifacts. While the C-terminus of perilipin 3 is stable in solution as an amphipathic α-helix bundle, the tertiary structure of the N-terminus is unknown but extended rather than compact, as judged from the x-ray scattering profile [65]. Attaching a highly hydrophilic β barrel protein such as GFP to the helix bundle domain may render this construct almost entirely cytosolic. In contrast, the same experiment with the 11-mer repeat region of the protein may lead to constructs that retain significant LD binding. We thus propose that the intracellular (*in vivo*) targeting of LDs by perilipins be further explored using approaches that do not rely on large hydrophilic fluorescent molecules (GFP is about the same size as the C-terminal domain, approximately 27 kDa and 28 kDa respectively).

## 5. Conclusions

Our results to date show that LD protein binding in vivo is likely governed by the physical chemistry of the lipid component of the LD monolayer. We observe in vitro that for both full-length perilipin 3 and its C-terminal amphipathic α-helix bundle, a fully unsaturated PC monolayer allows for greater protein insertion at the oil-lipid-aqueous interface. Furthermore, we observe that the addition of PE increases insertion of this C-terminal domain, but not full-length perilipin 3, at the oil-phospholipid-interface. However, these results raise important questions as to how this specificity of binding is achieved, and whether other perilipins show similar or distinct behavior.

## Figures and Tables

**Table 1:** Examples of triolein droplet surface tension values as reported by ADSA.

Triolein Drop	15 μL [mN/m]	10 μL [mN/m}	5 μL [mN/m]
Neat Oil	37.9 ± 0.5	39.6 ± 0.3	64 ± 9
POPC	31.6 ± 0.4	30.4 ± 0.4	31.2 ± 1.5

**Table 2:** Maximum change in monolayer pressure on triolein drop, Δπ_MAX_, and maximum insertion pressure (MIP) data derived from Figure 5a for the C-terminal amphipathic α-helix bundle of perilipin 3. Uncertainty values represent 95% confidence intervals.

PLN3D	ΔπMAX (mN/m)	MIP (mN/m)
POPC	19.1 ± 2.0	31 ± 4
POPC/POPA	22.2 ± 2.5	29 ± 4
POPC/POPE	27 ± 3	37 ± 4
POPC/POG	22.1 ± 2.2	35 ± 4

**Table 3:** Maximum change in monolayer pressure on triolein drop, Δπ_MAX_, and MIP data derived from Figure 5b for the full-length perilipin 3 construct. Uncertainty values represent 95% confidence intervals.

PLN3A	ΔπMAX (mN/m)	MIP (mN/m)
POPC	25.8 ± 1.2	30 ± 3
POPC/POPA	26 ± 3	31 ± 4
POPC/POPE	27.3 ± 1.2	33 ± 4
POPC/POG	22.9 ± 2.3	26 ± 4

## Data Availability

Data is contained within the article or supplementary material. Raw data and metadata are available on request from the corresponding authors and will be curated according the Data Management Plan applicable to NSF grant CHE-1808281.

## Supplementary Materials

The following are available online at https://www.mdpi.com/article/10.3390/membranes11040265/s1.
