# Supplementary material for: The C-Terminus of Perilipin 3 Shows Distinct Lipid Binding at Phospholipid-Oil-Aqueous Interfaces
Source: Membranes (Basel). 2021 Apr 6;11(4):265. doi: 10.3390/membranes11040265 (PMC8067514; doi:10.3390/membranes11040265)
Supplement: Supplementary file 1 [file membranes-11-00265-s001.pdf]

membranes

The C-Terminus of Perilipin 3 Shows Distinct Lipid Binding at Phospholipid-Oil-Aqueous Interfaces

Amber R. Titus [1,*], Ellyse N. Ridgway [1], Rebecca Douglas [1], Elena Sánchez Brenes [3], Elizabeth K. Mann [2] and Edgar E. Kooijman [1,*]

1 Department of Biological Sciences, Kent State University, Kent, OH, 44242, USA; eridgwa1@kent.edu (E.N.R.); rdougl11@kent.edu (R.D.)
2 Department of Physics, Kent State University, Kent, OH, 44242, USA; emann@kent.edu
3 Escuela de Física, Universidad de Costa Rica, San José, 11501, Costa Rica; elena.sanchezbrenes@ucr.ac.cr
* Correspondence: atitus3@kent.edu (A.R.T.), ekooijma@kent.edu (E.E.K.)

1. Supplementary Materials

1.1 Perilipin 3 secondary structure analysis

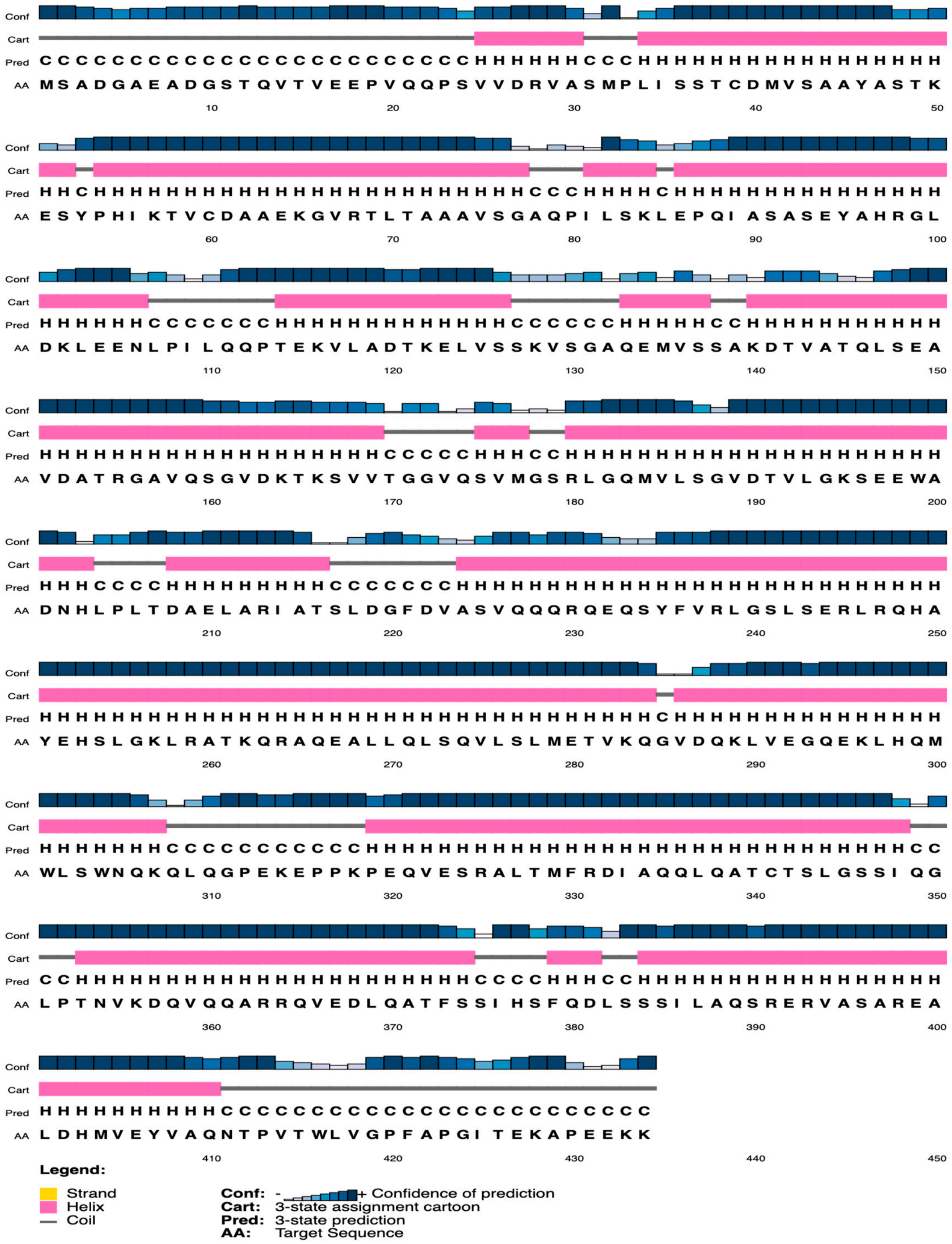

Supplementary Figure S1. PSIPRED (*1*) analysis of full-length perilipin 3. The large pink bars show predicted helix regions used for helical wheel generation.

1.2 Perilipin 3 11-mer region helical wheel representations

(a) Helix 1:

^{86}EPQIASASEYAHRGLDKLEEN$_{107}$

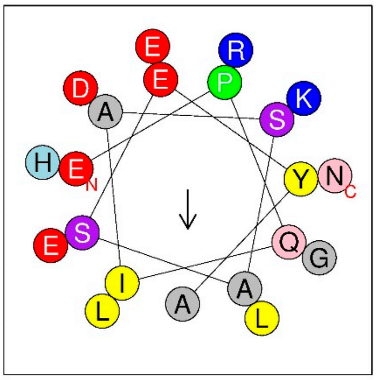

(b) Helix 2:

^{114}TEKVLADTKELVS$_{126}$

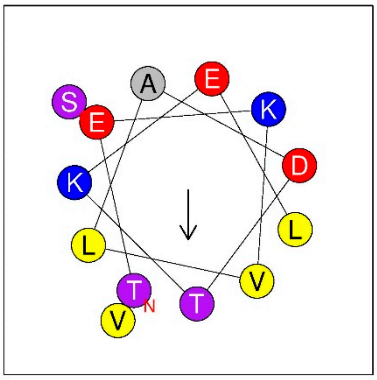

(c) Helix 3:

^{140}KDTVATQLSEAVDATRGAV QSGVDKTKSVV$_{169}$

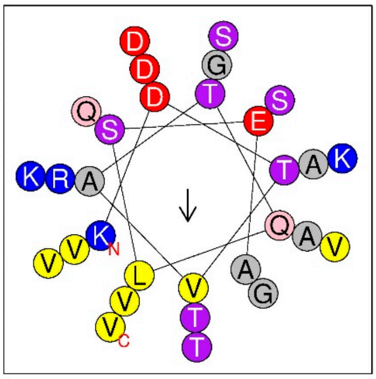

(d) Helix 4:

^{180}RLGQMVLSGVDTVLG KSEEWADNH$_{203}$

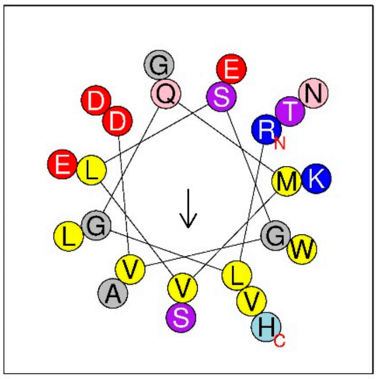

Supplementary Figure S2. Helical wheel representation of four predicted α helices in the 11-mer region of perilipin 3 generated using Heliquest (*2*).

	Net Charge	Hydrophobic Moment	Hydrophobicity
Helix 1	-3	0.337	0.081
Helix 2	-1	0.432	0.200
Helix 3	0	0.319	0.127
Helix 4	-2	0.386	0.301

Supplementary Table S1. The calculated net charge, hydrophobic moment, and hydrophobicity values of the helical wheel representations in Supplementary Figure 2 provided by Heliquest.

1.3 Perilipin 3 C-terminal α-helix bundle helical wheel representations

Data taken from Mirheydari et al., 2016 (*3*), and the crystal structure of the domain. These amphipathic helixes largely overlap with the predicted secondary structure using PSIPRED.

(a) Helix 1:

^{244}ERLRQHAYEHSLGKLRATKQ

RAQEALLQLSQALSLMETVK$_{283}$

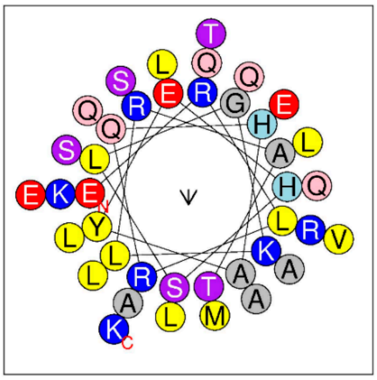

(b) Helix 2:

^{317}PKPEQVESRALTMFRD

IAQQLQATCTSLGSSI$_{349}$

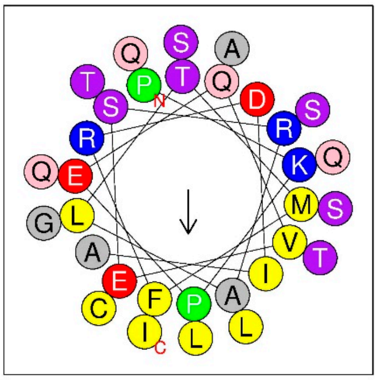

(c) Helix 3:

^{354}TNVKDQVQQARRQVEDL

QATF$_{373}$

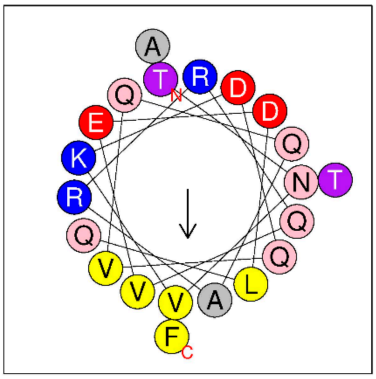

(d) Helix 4:

^{383}SSSILAQSRERVASAR

EALDHMVEYVAQ$_{412}$

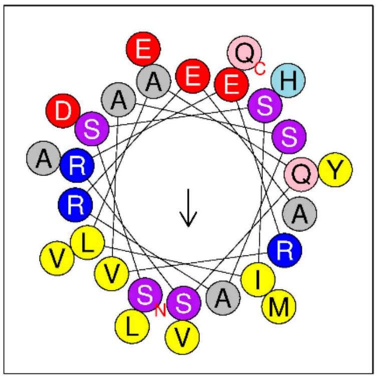

Supplementary Figure S3. Helical wheel representation of four predicted α helices in the C-terminal amphipathic α-helix region of perilipin 3 generated using Heliquest.

	Net Charge	Hydrophobic Moment	Hydrophobicity
Helix 1	5	0.381	0.360
Helix 2	0	0.373	0.354
Helix 3	0	0.425	0.067
Helix 4	0	0.321	0.227

Supplementary Table S2. The calculated net charge, hydrophobic moment, and hydrophobicity values of the helical wheel representations in Supplementary Figure 3 provided by Heliquest.

References

1. Buchan, D. W. A., and Jones, D. T. (2019) The PSIPRED Protein Analysis Workbench: 20 years on, *Nucleic Acids Res 47*, W402-W407.

2. Gautier, R., Douguet, D., Antonny, B., and Drin, G. (2008) HELIQUEST: a web server to screen sequences with specific alpha-helical properties, *Bioinformatics 24*, 2101-2102.

3. Mirheydari, M., Rathnayake, S. S., Frederick, H., Arhar, T., Mann, E. K., Cocklin, S., and Kooijman, E. E. (2016) Insertion of perilipin 3 into a glycero(phospho)lipid monolayer depends on lipid headgroup and acyl chain species, *J Lipid Res 57*, 1465-1476.
